# Impact of incomplete ventricular coverage on diagnostic performance of myocardial perfusion imaging

**DOI:** 10.1007/s10554-017-1265-1

**Published:** 2017-12-01

**Authors:** Behzad Sharif, Manish Motwani, Reza Arsanjani, Rohan Dharmakumar, Mathews B. Fish, Guido Germano, Debiao Li, Daniel S. Berman, Piotr Slomka

**Affiliations:** 1 Laboratory for Translational Imaging of Microcirculation, Biomedical Imaging Research Institute, Cedars-Sinai Medical Center, 8700 Beverly Blvd, Los Angeles, CA 90048 USA; 2 Biomedical Imaging Research Institute, Cedars-Sinai Medical Center, 8700 Beverly Blvd, Los Angeles, CA 90048 USA; 3 Department of Biomedical Sciences, Cedars-Sinai Medical Center, 8700 Beverly Blvd, Los Angeles, CA 90048 USA; 4 Departments of Imaging and Medicine, Cedars-Sinai Medical Center, 8700 Beverly Blvd, Los Angeles, CA 90048 USA; 5 David Geffen School of Medicine at UCLA, University of California Los Angeles, 757 Westwood Plaza, Los Angeles, CA 90095 USA; 6 Oregon Heart and Vascular Institute, Sacred Heart Medical Center, 3311 Riverbend Dr, Springfield, OR 97477 USA; 7 Division of Cardiovascular Medicine, Mayo Clinic, 13400 E Shea Blvd, Scottsdale, AZ 85259 USA

**Keywords:** Myocardial ischemia, Myocardial perfusion imaging, Cardiac magnetic resonance, Coronary artery disease, Myocardial ischemic burden, Whole heart imaging

## Abstract

In the context of myocardial perfusion imaging (MPI) with cardiac magnetic resonance (CMR), there is ongoing debate on the merits of using technically complex acquisition methods to achieve whole-heart spatial coverage, rather than conventional 3-slice acquisition. An adequately powered comparative study is difficult to achieve given the requirement for two separate stress CMR studies in each patient. The aim of this work is to draw relevant conclusions from SPECT MPI by comparing whole-heart versus simulated 3-slice coverage in a large existing dataset. SPECT data from 651 patients with suspected coronary artery disease who underwent invasive angiography were analyzed. A computational approach was designed to model 3-slice MPI by retrospective subsampling of whole- heart data. For both whole-heart and 3-slice approaches, the diagnostic performance and the stress total perfusion deficit (TPD) score—a measure of ischemia extent/severity—were quantified and compared. Diagnostic accuracy for the 3-slice and whole-heart approaches were similar (area under the curve: 0.843 vs. 0.855, respectively; *P* = 0.07). The majority (54%) of cases missed by 3-slice imaging had primarily apical ischemia. Whole-heart and 3-slice TPD scores were strongly correlated (R^2^ = 0.93, *P* < 0.001) but 3-slice TPD showed a small yet significant bias compared to whole-heart TPD (− 1.19%; *P* < 0.0001) and the 95% limits of agreement were relatively wide (− 6.65% to 4.27%). Incomplete ventricular coverage typically acquired in 3-slice CMR MPI does not significantly affect the diagnostic accuracy. However, 3-slice MPI may fail to detect severe apical ischemia and underestimate the extent/severity of perfusion defects. Our results suggest that caution is required when comparing the ischemic burden between 3-slice and whole-heart datasets, and corroborate the need to establish prognostic thresholds specific to each approach.

## Introduction

Direct visualization of perfusion abnormalities using stress myocardial perfusion imaging (MPI) enables noninvasive assessment of the functional relevance of impaired coronary blood flow, and is currently the preferred test in patients with suspected ischemic heart disease. With several million studies performed in the U.S. alone, myocardial perfusion is most commonly assessed using nuclear imaging modalities coupled with stress-induced hyperemia to detect hypoperfusion [1]. Within the realm of single-photon emission computed tomography (SPECT) MPI, various studies have documented an important prognostic significance associated with stress-induced myocardial perfusion deficits [2, 3]. With recent technical improvements, first-pass MPI using cardiac magnetic resonance (CMR) is emerging as an alternative to SPECT MPI [4–6]. Recent single-and multi-center clinical trials have shown non-inferiority of CMR MPI in detecting significant coronary artery disease (CAD) in comparison to SPECT [7–9].

Despite recent developments, CMR MPI remains as one of the most technically challenging modalities for detection of ischemia and achieves a limited spatial coverage of the left ventricle, typically 3 short-axis slices: basal, mid ventricular, and apical [10, 11]. Among the most significant recent technical advances for CMR MPI is the ability to achieve whole-heart spatial coverage [12–21]. Whole-heart CMR MPI has notable advantages relative to the conventional 3-slice CMR—specifically, the ability to yield the myocardial ischemic burden as a potentially important prognostic index similar to nuclear MPI methods [16–19]. However, unlike nuclear MPI methods, which intrinsically achieve whole-heart coverage, extending the spatial coverage to whole-heart in CMR methods involves technical challenges, advanced computational platforms and image-quality tradeoffs [14–19].

Weighing such potential advantages against current technical challenges, there is ongoing debate on the merits of whole-heart CMR MPI compared with conventional 3-slice imaging in terms of improving the overall diagnostic performance and assessment of extent/severity of ischemia [20, 21]. Within the same context and considering the growing number of clinical studies comparing nuclear MPI and 3-slice CMR MPI [7–9], it is not clear to what extent nuclear MPI methods benefit from their inherent whole-heart imaging capability, or how assessments of ischemic burden vary between the two modalities with such different levels of spatial coverage. The purpose of this study is to compare the diagnostic performance of whole-heart and 3-slice MPI and their assessment of the extent/severity of stress-induced perfusion deficits, using nuclear MPI as the modality being tested. To this end, our study is based on retrospective analysis of a large SPECT MPI dataset with correlative invasive coronary angiography (ICA), and the results are discussed in terms of their implication with respect to CMR protocols.

## Materials and methods

### Studied population

Subjects were selected from 9709 patients with suspected CAD who were referred for a stress/rest SPECT MPI study from 2003 to 2006 [22]. The SPECT MPI study protocols for retrospective analysis of anonymized data were approved by the local institutional review board. The exclusion criteria for the selected patients included prior history of CAD including prior myocardial infarction, cardiomyopathy, significant valve disease, left bundle branch block, and paced rhythm. The studied population was limited to patients who had also undergone correlative ICA performed within 60 days of imaging with no intervening event. A total of 651 sequential studies were retrospectively identified to form the studied population. Informed consent was obtained from all individual participants included in the study.

### Imaging protocol

Subjects underwent electrocardiography-gated SPECT MPI using a standard ^99m^Tc-sestamibi rest/stress protocol. Rest images were acquired 60 min after administration of the radiotracer. This was followed by stress imaging performed at 15–45 min after either adenosine infusion with low-level exercise or radiopharmaceutical injection during treadmill exercise stress. Dual-detector scintillation cameras (Vertex, Philips Medical Systems, Milpitas, CA) were used. Image acquisition and tomographic reconstruction were performed using the previously described conventional protocol [23]. Gender-matched normal limits for MPI were employed, derived from a separate group of 100 subjects (50 males, 50 females) with low likelihood of CAD and visually normal scans [23].

### Data analysis methods

#### Method for automatic analysis of myocardial perfusion studies

**Fig. 1:**
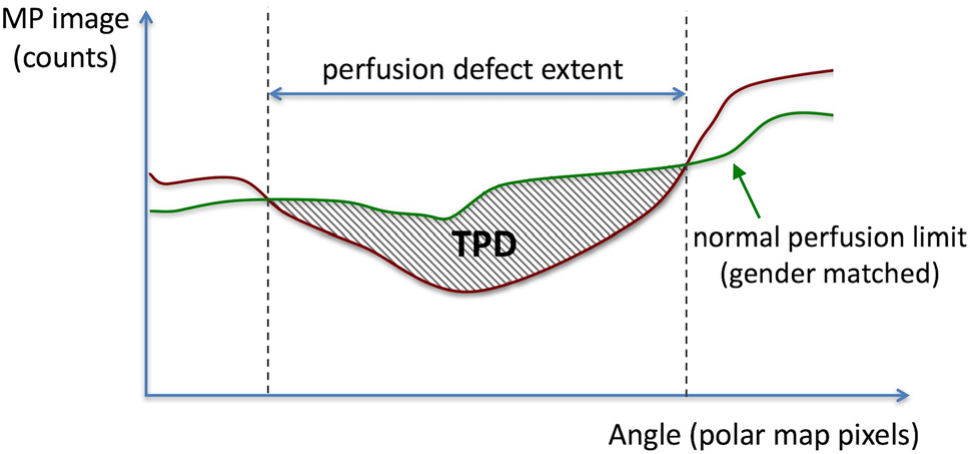
Schematic description of the total perfusion deficit (TPD) score for analysis of SPECT myocardial perfusion imaging (MPI). TPD is a continuous measure of the combined extent and severity of perfusion defects, designed to be equivalent to the visual summed segmental score. Derived automatically from SPECT MPI data using the Quantitative Perfusion SPECT (QPS) software, presence and severity of stress-induced hypoperfusion is measured relative to gender-matched normal perfusion limits for each pixel on the polar map (using a mean absolute deviation threshold of 3.0). Whole-heart stress TPD is computed by integrating the stress hypoperfusion severities across the left ventricular polar map, expressed in a percentage format

MPI images were automatically analyzed based on the clinically validated measure of total perfusion deficit (TPD) [24, 25] using the standard Quantitative Perfusion SPECT algorithm (QPS software, version 2013, Cedars-Sinai) [26, 27]. TPD is a continuous measure of perfusion defect extent and severity, designed to be equivalent to the commonly used summed segmental scoring [24, 25]. Figure 1 provides a schematic description of the TPD score. To compute the TPD for a given patient, in the first step a standard ellipsoidal contouring model for the left ventricular (LV) surface was automatically generated in the QPS software. Polar-map samples were extracted from the stress SPECT MPI data corresponding to the maximum count profiles normal to the LV surface [26]. Next, for each pixel on the polar map, presence of hypoperfusion was detected by comparison to gender-matched normal perfusion limits using a mean absolute deviation threshold of 3.0 (equivalent to ≈ 2.5 standard deviation) [23]. Finally, severity of hypoperfusion among the abnormal polar-map pixels (detected in the previous step) was computed and integrated to yield the “whole-heart TPD,” which is expressed in a percentage format (with a theoretical maximum of 100% corresponding to no visible uptake in the entire LV surface). Previous studies have established that automated evaluation of SPECT MPI using the described stress TPD measure achieves a diagnostic performance equivalent to, or better than, expert visual analysis [24, 28].

#### Method for retrospective simulation of 3-slice perfusion imaging

**Fig. 2:**
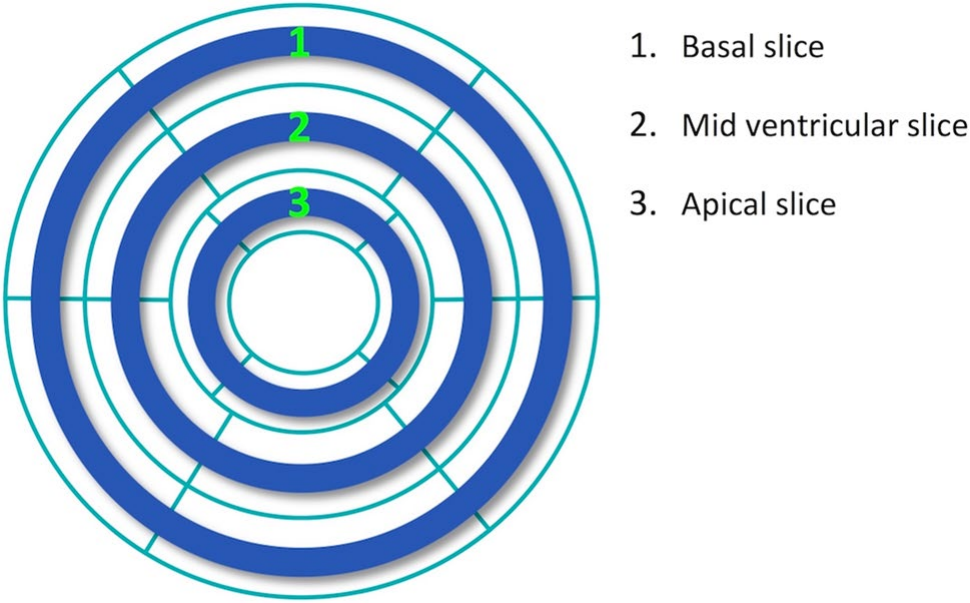
Demonstration of the 3-slice binary mask (blue color) superimposed on the whole-heart LV polar map used in the QPS software for retrospective analysis of SPECT MPI data. Two consecutive rings from the whole-heart polar map in the QPS software were combined (thickness ≈ 10 mm) at the center of apical, mid ventricular, and basal positions (defined by the AHA 17-segment LV model), respectively. To simulate 3-slice imaging using the SPECT MPI datasets, this binary mask was applied to the whole-heart data (polar map) in order to select only 3 short-axis slices as conventionally acquired in magnetic resonance MPI (apical, mid ventricular, and basal slices). *LV* left ventricular, *MPI* myocardial perfusion imaging. (Color figure online)

We developed a computational approach for retrospective analysis of the whole-heart MPI dataset to model/simulate 3-slice MPI. The approach was based on a modified TPD metric and leveraged the automatic perfusion-assessment capability and flexibility of the QPS software. Briefly, a new set of truncated “3-slice polar maps” was derived from the set of SPECT MPI whole-heart polar maps corresponding to the 651 studied patients. First, for a given patient, a binary mask corresponding to the whole-heart polar map was generated that selected only 3 short-axis slices at apical, mid ventricular, and basal position—same 3 slices conventionally acquired in CMR MPI [10, 11]. Specifically, as demonstrated in Fig. 2, two consecutive rings from the whole-heart polar map used in the QPS software were combined (≈ 10 mm slice thickness) at the center of apical, mid ventricular, and basal positions as defined by the AHA 17-segment LV model [29]. Next, this 3-slice binary mask was applied to the corresponding whole-heart polar maps (stress images) generating the 3-slice polar map, which subsampled the whole-heart data down to 3 short-axis slices. Finally, the new measure of “3-slice TPD” was computed according to the same formulation as whole-heart TPD but only considering hypoperfusion severity/extent within the 3-slice polar map, and expressed as a normalized percentage. Analysis was performed in completely automated mode using the same LV contour definitions as obtained for whole-heart MPI.

### Statistical methods

Based on the ICA data, significant CAD was defined as a coronary stenosis of ≥ 70% on visual assessment in any of the main epicardial coronary arteries or their branches with diameter ≥ 2 mm. Receiver-operating characteristic (ROC) analysis was used to determine the diagnostic performance (area under the curve [AUC]) for each of the TPD measures (whole-heart and 3-slice) and these were compared according to the Delong method [30]. Sensitivity and specificity of both techniques were compared using McNemar’s paired test. The optimal cut-off threshold for whole-heart stress TPD was ≥ 3% based on a method described in prior studies [22]. For the 3-slice technique, the optimal TPD cut-off threshold was determined by the same method and was also ≥ 3% on a per-patient basis. Agreement and correlation between the two TPD measures (whole-heart vs. 3-slice) was evaluated in patients with significant CAD using linear regression and Bland–Altman analysis [31]. The categorization of TPD as < 3% or ≥ 3% by both techniques was also compared. All statistical tests were 2-tailed and p < 0.05 was considered significant. All statistical calculations were performed using Analyse-It version 2.10 (Analyse-it Software Ltd., Leeds, UK).

## Results

### Study population

Characteristics of the studied patient population are summarized in Table 1. Of the 651 patients, 187 (29%) had no significant CAD based on ICA, and the rest (n = 464) comprised a relatively even mixture of single-vessel (36%) and multi-vessel (30%) disease with “vessel” referring to the left main artery or one of the three major epicardial arteries: left anterior descending artery (LAD), left circumflex artery, or right coronary artery. Furthermore, of the 464 patients with significant CAD based on ICA, 35 patients (5%) did not have a significant stenosis in any of their major epicardial arteries and instead had ≥ 70% stenosis in one or more of the diagonal/obtuse marginal branches, or the posterior descending artery.

**Table 1:** Characteristics of the studied patient population

Characteristic	Patients
Number	651
Age (years)	64 ± 12
Male (%)	57
Female (%)	43
Mean body mass index ± SD	31 ± 6.3
Diabetes mellitus (%)	27
Hypertension (%)	64
Hyperlipidemia (%)	51
Smoking (%)	19
Exercise SPECT (%)	34
Adenosine SPECT (%)	66
Mean ejection fraction ± SD (%)	61.7 ± 12.2
ICA results (disease defined as ≥ 70% stenosis)	
No disease	187 (29%)
LAD disease	280 (43%)
LCX disease	169 (26%)
RCA disease	247 (38%)
1-vessel disease^‡^	232 (36%)
2-vessel disease^‡^	127 (19%)
3-vessel disease^‡^	70 (11%)

A total of 651 SPECT myocardial perfusion imaging studies were analyzed. All patients underwent correlative invasive coronary angiography (ICA)

*LAD* left anterior descending artery, *LCX* left circumflex artery, *RCA* right coronary artery, *SD* standard deviation, *SPECT* single-photon emission computed tomography

^‡^Vessel refers to one of the major epicardial arteries (left main, LAD, LCX, or RCA)

### Diagnostic accuracy

**Fig. 3:**
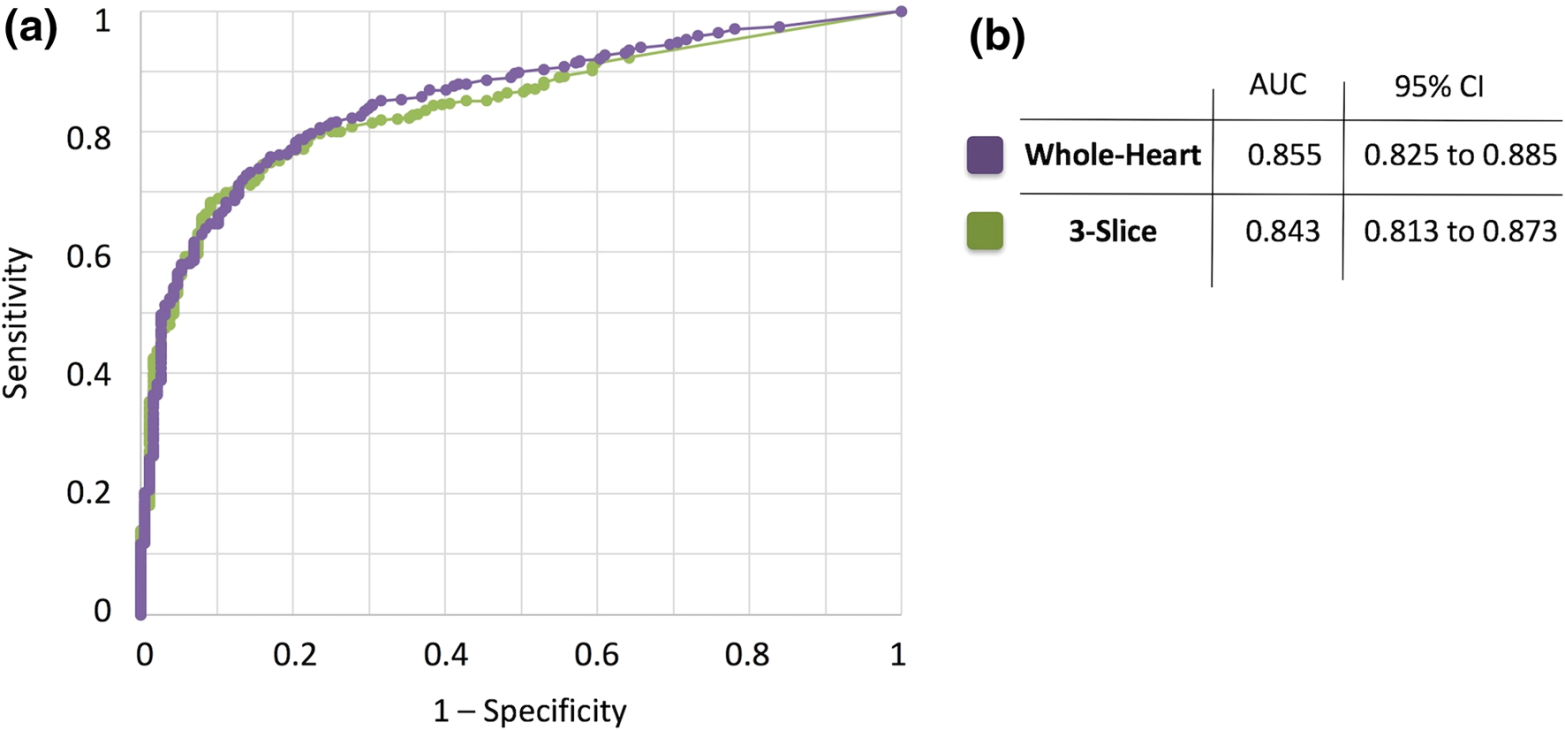
Comparison of the diagnostic accuracy of whole-heart myocardial perfusion imaging (MPI) versus 3-slice MPI with invasive coronary angiography (ICA) as the reference. **a** Receiver-operating characteristic (ROC) curves corresponding to whole-heart imaging (using the whole-heart TPD scores) versus 3-slice imaging (using the 3-slice TPD scores) for detection of significant CAD, defined as ≥ 70% coronary stenosis (any main epicardial vessel or branches with diameter ≥ 2 mm) based on ICA (n = 651). **b** The area under the ROC curves (AUCs) for whole-heart imaging and 3-slice imaging are 0.855 and 0.843, respectively (*P* = 0.07), indicating a statistically insignificant difference in terms of diagnostic performance. The 95% confidence interval corresponding to each AUC is also provided in panel (**b**). *CAD* coronary artery disease, *TPD* total perfusion deficit

To directly compare the diagnostic accuracy of whole-heart and 3-slice imaging, we generated ROC curves for detection of significant CAD for each approach as shown in Fig. 3a. As described in Fig. 3b, the AUC for whole-heart imaging and 3-slice imaging were similar with no significant difference (0.855 ± 0.015 vs. 0.843 ± 0.015, respectively; *P* = 0.07). At the optimal TPD cut-off threshold (≥ 3% for both techniques), whole-heart imaging had a higher sensitivity than 3-slice imaging (84.5% vs. 80.1%, respectively; *P* < 0.01) but specificities showed no significant difference (70.0% vs. 74.9%, respectively; *P* = 0.08).

### TPD scores: level of agreement

**Fig. 4:**
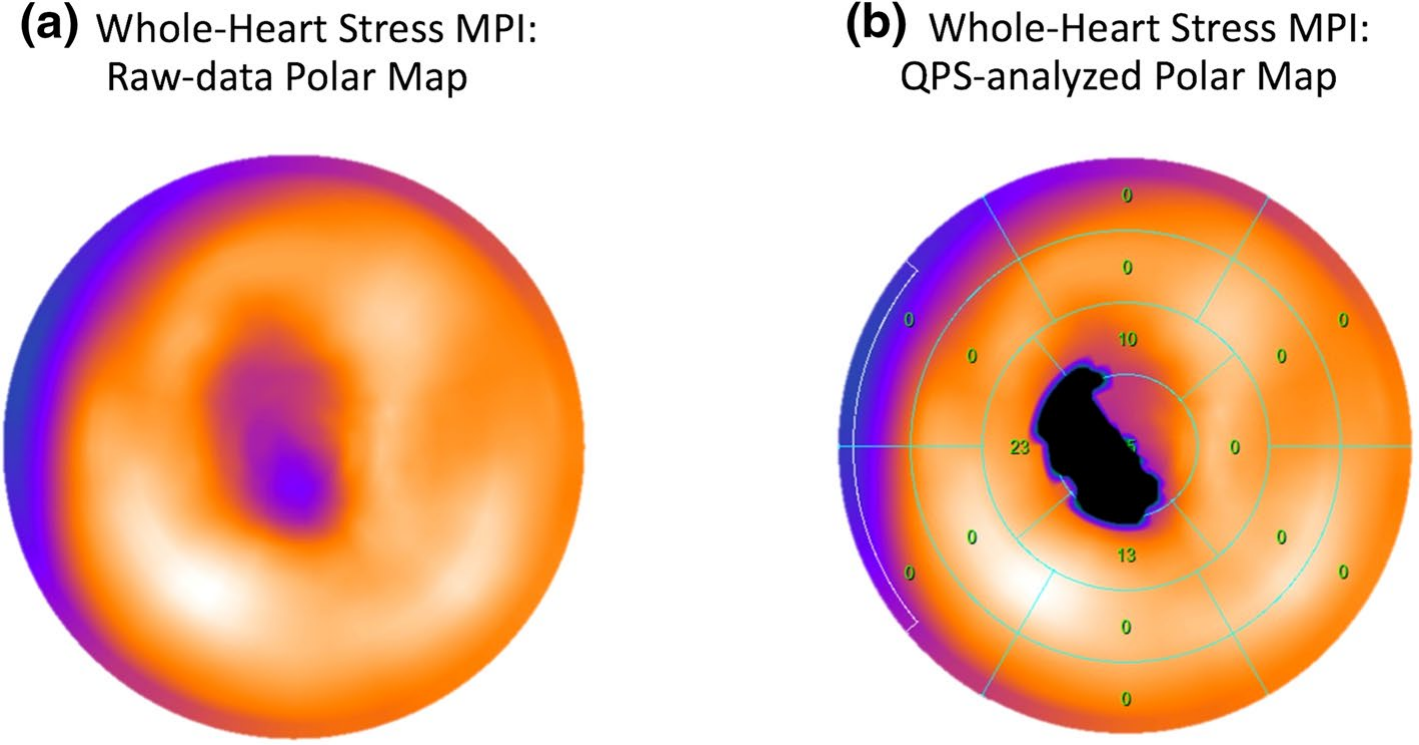
Example stress SPECT MPI data for a case wherein ischemia is detected on the basis of the whole-heart TPD score but 3-slice TPD measurement fails to detect the severe ischemia. **a** Polar map with raw perfusion data; **b** automatically analyzed whole-heart polar maps generated in the QPS software (whole-heart stress TPD = 6.0%). The presented case is the stress MPI data for a 58-year-old female patient showing a severe apical perfusion defect, which is consistent with her invasive angiography results that indicated 90% LAD stenosis. In **b**, the measured perfusion defect region is shown in black and the numbers in each segment indicate the corresponding defect extent (in percentage) for that segment. The 3-slice stress TPD (computed from the conventional short-axis slice positions as shown in Fig. 2) was 0.9%, which is below the 3% abnormality threshold. *LAD* left anterior descending, *MPI* myocardial perfusion imaging, *TPD* total perfusion deficit

Among the 464 patients with significant CAD based on ICA, TPD was found to be ≥ 3% by both methods in 367 patients; and < 3% by both methods in 67 patients. Overall, when used to categorize TPD as ≥ 3% or < 3%, there was excellent agreement between the two techniques (observed agreement = 93.5%). In 30 patients with significant CAD, the two methods disagreed at the ≥ 3% threshold: in 26 patients, the whole-heart method determined TPD ≥ 3% (true positives) but 3-slice stress TPD estimated it as < 3% (mean TPD difference = 3.2%); and in 4 patients, the whole-heart method estimated TPD as < 3%, but 3 slice technique estimated it as ≥ 3% (mean TPD difference = 0.9%). Figure 4 shows an example of such a case of disagreement in which ischemia is detected on the basis of whole-heart TPD but 3-slice TPD fails to detect the perfusion defect since it is located at the apex (details provided in caption). Overall, in 14 of the 26 cases that were detected by whole-heart but missed by 3-slice, the perfusion defect was mainly apical (similar to Fig. 4) and corresponded to significant single-vessel LAD disease on ICA.

**Fig. 5:**
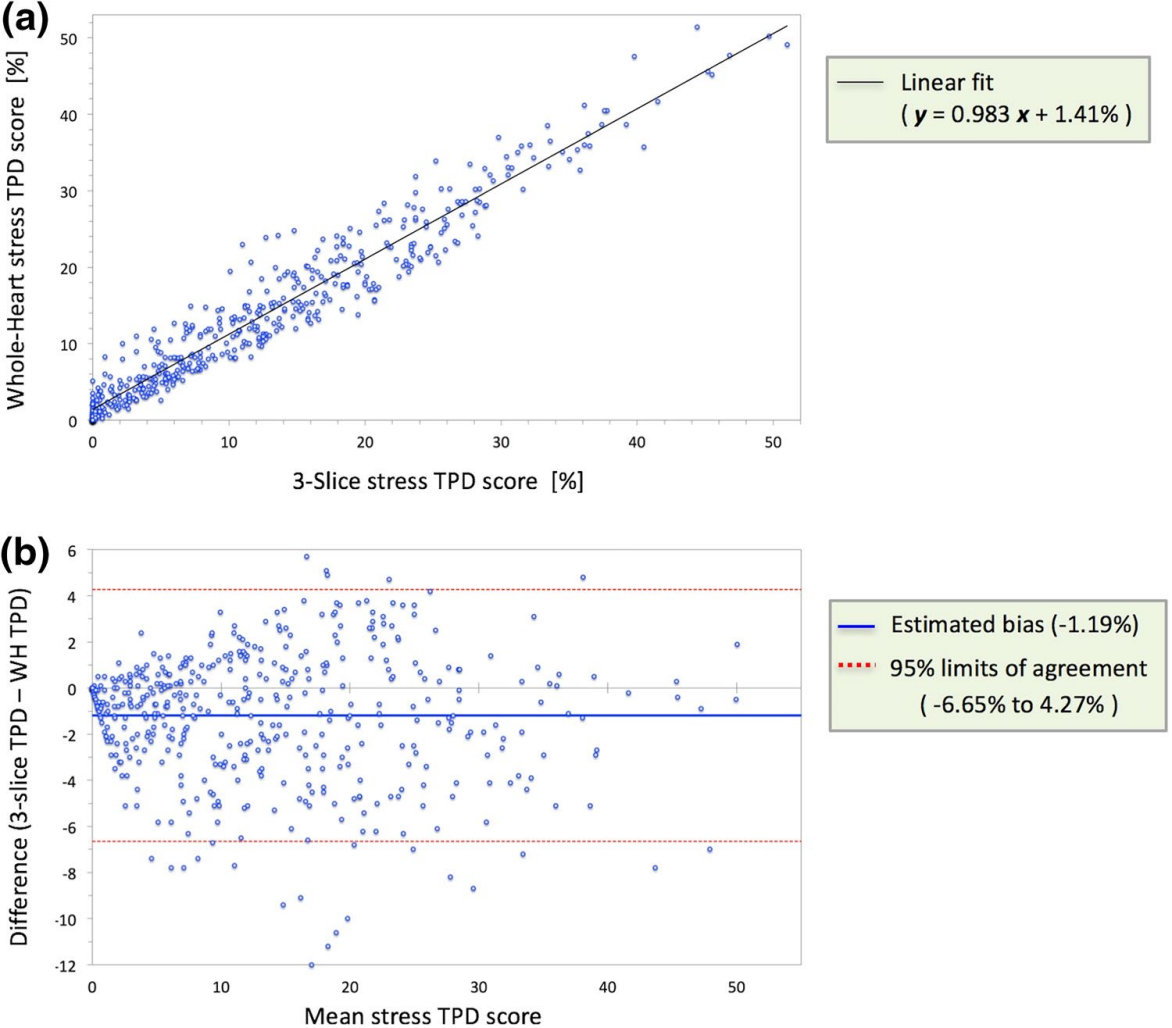
Evaluation of correlation and agreement between whole-heart versus 3-slice stress TPD scores in patients with significant CAD (≥ 70% stenosis; n = 464). **a** Scatter plot and linear regression of whole-heart TPD against 3-slice TPD showing a very strong correlation (R^2^ = 0.93, *P* < 0.001) but a noticeable intercept of 1.41% (95% CI: 1.01% to 1.80%) indicating the presence of a systematic bias. **b** Bland–Altman analysis demonstrates a moderate level of agreement between whole-heart and 3-slice TPD scores that decreases at higher TPD values (95% limits of agreement: − 6.65% to 4.27%), and a small but significant systematic bias of − 1.19% (*P* < 0.0001; 95% CI: − 1.45% to − 0.94%). *CAD* coronary artery disease, *CI* confidence interval, *TPD* total perfusion deficit

Linear regression analysis in patients with significant CAD (n = 464), shown in Fig. 5a, demonstrated very strong correlation between whole-heart and 3-slice TPD scores (R^2^ = 0.93, *P* < 0.001) but with a noticeable intercept of 1.41% (95% confidence interval (CI): 1.01% to 1.80%). Bland–Altman analysis in patients with significant CAD, shown in Fig. 5b, demonstrated good agreement at low TPD values but only a moderate level of agreement was observed for the medium-to-high range of TPD scores (mean TPD ≥ 8%). Overall, the 95% limits of agreement were − 6.65% to 4.27% and the estimated bias was − 1.19% (*P* < 0.0001; 95% CI: − 1.45% to − 0.94%).

## Discussion

Despite having a limited spatial coverage of the LV myocardium, assessment of myocardial perfusion using vasodilator stress CMR has been shown to be an effective diagnostic tool in evaluation of patients with suspected CAD, and yet it remains as one of the most technically challenging modalities for MPI. Whole-heart CMR MPI is even more challenging and is accompanied with higher likelihood of artefactual perfusion images, especially due to high heart rates or inability of the patient to perform a breath-hold during the stress MPI scan [21, 32, 33]. Compared to the conventional 3-slice CMR MPI approach, however, whole-heart CMR techniques have notable advantages including accurate assessment of ischemic burden and detection of apical ischemia, and are therefore being extensively developed [14–19]. In fact, a recent multi-center study showed excellent diagnostic performance for whole-heart CMR MPI in detecting significant CAD [34]. On the other hand, conventional 3-slice MPI has also been shown to achieve high diagnostic accuracy in large single- and multi-center clinical trials without the additional technical complexity of the whole-heart approach [7–9]. Furthermore, whole-heart CMR MPI requires a high “imaging acceleration” factor (as high as 8–10 fold [21]) and therefore involves inevitable trade-offs in terms of spatial and temporal fidelity (loss of high-frequency information) and/or image quality—specifically, reduced in-plane resolution and a larger acquisition window (temporal footprint) within each cardiac cycle, which both may contribute to increased prevalence/frequency of image artifacts. Consequently, there is ongoing debate on the optimal technique for CMR MPI protocols and a need to better understand the impact of spatial coverage on diagnostic performance of MPI [20, 21].

Ultimately, a large-scale study evaluating both whole-heart and 3-slice stress CMR MPI in the same patient population can provide a definitive assessment of the merits of whole-heart CMR. *Although this is the ideal approach, a large study population would be required to detect a discernible difference with adequate power—as both techniques are already known to have high diagnostic accuracy*. Furthermore, recruitment would be challenging given the requirement for *two serial stress CMR exams for each patient*. An alternative to performing an adequately-powered comparative CMR study is to draw relevant conclusions from a large existing nuclear MPI dataset. Therefore, in the current study, we used a computational approach to systematically study the effect of reduced spatial coverage on the diagnostic performance of MPI using a large SPECT dataset with available correlative invasive angiography. This computational data analysis was enabled by an automated measure of stress-induced perfusion defect extent and severity, namely, the TPD score, using a previously established approach shown to be equivalent to the standard visual assessment.

### Main findings

*The first finding of this study*, based on retrospective analysis of a SPECT MPI dataset, is that the diagnostic accuracy of MPI with whole-heart spatial coverage is similar to MPI with 3-slice coverage (distributed at basal, mid ventricular, and apical positions with ≈ 10 mm thickness) for detection of significant CAD (AUC: 0.855 vs. 0.843 respectively; *P* = 0.07). This finding is in keeping with a smaller CMR-based study (n = 53) by Jogiya et al. [19], which took a similar approach with CMR and also found no difference in diagnostic accuracy between whole-heart acquisition and a 3-slice model selected from the same CMR data. The limitations of the Jogiya et al. study were its small sample size (53 vs. 651 in our study) and a potentially unfair representation of the 3-slice technique as noted in the same study [19]. (Image quality of the selected 3 slices from whole-heart CMR data was inferior to separately-acquired 3-slice data because of the inherent CMR data acquisition trade-offs involving spatial and temporal resolution [21]). In this context, our study is particularly additive as it overcomes both of these limitations by using a large nuclear MPI dataset.

*The second finding of the current study* is that a majority (54%) of the cases missed by 3-slice MPI involved patients with significant single-vessel LAD disease for which ischemia was mainly located at the apex (17th segment in the AHA myocardial segmentation model), which is not adequately captured by 3-slice imaging. Consequently, our results suggest that, in CMR protocols, potential diagnostic benefits of whole-heart imaging will be due to the ability to capture apical defects in patients with LAD disease. This phenomenon may also explain the systematic underestimation of TPD (significant negative bias) with 3-slice coverage compared to whole-heart MPI (bias: − 1.19%; *P* < 0.0001). A prudent approach for overcoming this limitation of 3-slice CMR MPI versus whole-heart imaging is to increase the ventricular coverage to 4 slices by adding a long-axis slice (2- or 4-chamber view) to the 3 short-axis slices. This approach will provide a notably improved visualization of the apical region and therefore may improve the detection of stress-induced perfusion defects that are primarily located at the apex. It should be noted, however, that addition of a 4th slice to a two-dimensional multi-slice CMR perfusion pulse sequence will inevitably limit the peak stress heart-rate than can be accommodated during acquisition of the first-pass of the contrast agent. In such patients, a potential solution for performing 4-slice CMR MPI is to employ a high level of parallel-imaging acceleration (beyond the typical twofold acceleration), which may result in reduced image quality.

*The third important finding* is that although there is a good correlation between the stress TPD scores for 3-slice and whole-heart imaging, there are relatively wide limits of agreement between the two TPD scores. Since our study population excluded patients with prior history of CAD and prior myocardial infarction, the stress TPD score presented in our results is a surrogate of ischemic burden. In this context, the Bland–Altman 95% agreement limits of − 6.65% to 4.27% are relatively wide considering that an ischemic threshold of 10% is often used to guide revascularization versus medical therapy decisions in whole-heart MPI [2, 3]. This implies that the “ischemic burden” derived/extrapolated from 3-slice imaging (e.g., those derived in clinical CMR studies) may not be an accurate measure for guiding therapy or assessment of risk in CAD patients based on the previously established cut-offs for whole-heart SPECT MPI. Our TPD-comparison results corroborate a recent pilot CMR MPI study of 27 subjects by McDiarmid et al. [35], which found a strong correlation but wide limits of agreement for estimates of ischemic burden between 3-slice and whole-heart CMR approaches. Notably, the latter pilot study called for a much larger study to confirm these findings but accepted the impracticality and significant cost given the requirement for two serial stress CMR MPI studies and an adequately-powered sample size [35]. Our study, although drawing on data from SPECT, has the significant advantage of a much larger study population (651 vs. 27)—enabling it to draw statistically meaningful conclusions.

### Study limitations

The presented work is a “simulation” study in that SPECT MPI inherently achieves whole-heart coverage and 3-slice imaging was simulated by subsampling of the whole-heart dataset. *Since the underlying data-acquisition physics and resolution of CMR and SPECT MPI are different (first pass of MR contrast agent vs. accumulation of the SPECT radio-tracer), we acknowledge that our results present a partial view into the potential advantages of whole-heart versus 3-slice CMR MPI*. However, a recent study has demonstrated a close agreement between ischemic burden identified by SPECT MPI and 3-dimensional whole-heart CMR MPI [36] and a similar but weaker agreement with SPECT MPI is likely to hold for 2-dimensional multi-slice CMR MPI, especially if “all systolic” methods are used [37]. Second, given the fully-automatic processing methods employed for quantification of TPD in our study, it can be argued that our comparison of the ischemic-burden surrogate (i.e., TPD) was free of “operator bias” and other complicating factors encountered in analysis of CMR MPI data, which often involves manual steps. In common with the majority of prior MPI studies, the use of a visually assessed anatomical reference standard for CAD is a known limitation, and fractional flow reserve would have been preferable. *However, this limitation is less relevant here as the same patients provide the two compared imaging methods from the same dataset, and therefore the deficiencies in ICA as a reference standard for ischemia affect both groups to a similar extent*.

## Conclusions

There is ongoing debate on the merits of using technically complex whole-heart MPI methods using cardiac magnetic resonance (CMR) rather than conventional 3-slice CMR acquisition. Based on retrospective analysis of a large SPECT MPI dataset, our results demonstrate that MPI with whole-heart coverage versus three short-axis slices—as typically acquired in CMR MPI protocols—have similar diagnostic accuracy for detection of significant CAD, defined as ≥ 70% coronary stenosis using invasive angiography. However, a detailed comparison of whole-heart versus 3-slice MPI indicates that 3-slice coverage may fail to detect severe ischemia that is primarily apical and can lead to a systematic underestimation of the TPD score—a surrogate of myocardial ischemic burden. The presented results suggest caution when comparing the myocardial ischemic burden between approaches with different spatial coverage (3-slice vs. whole-heart datasets), and therefore underlines the need to establish prognostic ischemic-burden thresholds specific to level of spatial coverage in CMR MPI protocols.

## Abbreviations

AUC: Area under the ROC curve
CAD: Coronary artery disease
CI: Confidence interval
CMR: Cardiac magnetic resonance
ICA: Invasive coronary angiography
LV: Left ventricular
QPS: Quantitative perfusion SPECT
ROC: Receiver-operating characteristic
TPD: Total perfusion deficit
